# Supplementary material for: Empirical methods for controlling false positives and estimating confidence in ChIP-Seq peaks
Source: BMC Bioinformatics. 2008 Dec 5;9:523. doi: 10.1186/1471-2105-9-523 (PMC2628906; doi:10.1186/1471-2105-9-523)
Supplement: Additional File 1 — A variety of html documents from the USeq web site detailing the available applications, their best usage, output file type descriptions, command line menus, etc. [file 1471-2105-9-523-S1.zip › USeqUserGuides/JavaDoc.zip!/JavaDoc/bioroot/UserRegistrationBean.html]

UserRegistrationBean


|  |  |  |
| --- | --- | --- |
| **Overview** \| **Package** \| **Class** \| **Use** \| **Tree** \| **Deprecated** \| **Index** \| **Help** | |  |
| **PREV CLASS**   **NEXT CLASS** | **FRAMES**    **NO FRAMES**     **All Classes** |  |
| SUMMARY: NESTED \| FIELD \| CONSTR \| METHOD | DETAIL: FIELD \| CONSTR \| METHOD |  |


---


## bioroot Class UserRegistrationBean

```
java.lang.Object
  bioroot.UserRegistrationBean
```

---

``` public class UserRegistrationBean extends java.lang.Object ```

**Author:**
:   nix
    Bean to hold UserRegistration data

---

**Constructor Summary**

`UserRegistrationBean()`

`UserRegistrationBean(UserBean user)`


| **Method Summary** | |
| --- | --- |
| `void` | `addOldUserAttributes()` |
| `boolean` | `checkParams()` |
| `boolean` | `checkUpdateParams(java.lang.String oldLoginName)`             Used when checking params for AccountManagement Update |
| `java.lang.String` | `extractType()` |
| `DBUtil` | `getBioroot()` |
| `java.lang.String` | `getDifferentUserConcat()` |
| `java.lang.String` | `getEmail()` |
| `java.lang.String` | `getFirstName()` |
| `java.lang.String` | `getLabGroup()` |
| `java.lang.String` | `getLastName()` |
| `java.lang.String` | `getLoginName()` |
| `java.lang.String` | `getMessages()` |
| `java.lang.String` | `getPassword1()` |
| `java.lang.String` | `getPassword2()` |
| `java.lang.String` | `getPhone()` |
| `UserBean` | `getSubmitterUserBean()` |
| `java.lang.String` | `getType()` |
| `int` | `getUserId()` |
| `boolean` | `isComplete()` |
| `boolean` | `isExistingMember()` |
| `boolean` | `isLoginNameUnique()`             checks to see if login name is unique, assumes a connection has been made |
| `boolean` | `isPasswordReset()` |
| `void` | `setBioroot(DBUtil util)` |
| `void` | `setComplete(boolean b)` |
| `void` | `setDifferentUserConcat(java.lang.String differentUserConcat)` |
| `void` | `setEmail(java.lang.String string)` |
| `void` | `setExistingMember(boolean existingMember)` |
| `void` | `setFirstName(java.lang.String string)` |
| `void` | `setLabGroup(java.lang.String string)` |
| `void` | `setLastName(java.lang.String string)` |
| `void` | `setLoginName(java.lang.String string)` |
| `void` | `setMessages(java.lang.String string)` |
| `void` | `setPassword1(java.lang.String string)` |
| `void` | `setPassword2(java.lang.String string)` |
| `void` | `setPasswordReset(boolean passwordReset)` |
| `void` | `setPhone(java.lang.String string)` |
| `void` | `setSubmitterUserBean(UserBean bean)` |
| `void` | `setType(java.lang.String string)` |
| `void` | `setUserId(int userId)` |
| `boolean` | `submitNewUser()` |
| `boolean` | `updateUser()` |
| `boolean` | `updateUserLabGroupTable(int userId, int labGroupId, java.lang.String type)` |

**Methods inherited from class java.lang.Object**

`equals, getClass, hashCode, notify, notifyAll, toString, wait, wait, wait`

**Constructor Detail**

### UserRegistrationBean

```
public UserRegistrationBean()
```

---


### UserRegistrationBean

```
public UserRegistrationBean(UserBean user)
```


**Method Detail**

### addOldUserAttributes

```
public void addOldUserAttributes()
```

---


### checkParams

```
public boolean checkParams()
```

---


### checkUpdateParams

```
public boolean checkUpdateParams(java.lang.String oldLoginName)
```

:   Used when checking params for AccountManagement Update

---


### isLoginNameUnique

```
public boolean isLoginNameUnique()
```

:   checks to see if login name is unique, assumes a connection has been made

---


### submitNewUser

```
public boolean submitNewUser()
```

---


### extractType

```
public java.lang.String extractType()
```

---


### updateUserLabGroupTable

```
public boolean updateUserLabGroupTable(int userId,
                                       int labGroupId,
                                       java.lang.String type)
```

---


### updateUser

```
public boolean updateUser()
```

---


### getEmail

```
public java.lang.String getEmail()
```

---


### getFirstName

```
public java.lang.String getFirstName()
```

---


### getLastName

```
public java.lang.String getLastName()
```

---


### getLoginName

```
public java.lang.String getLoginName()
```

---


### getPhone

```
public java.lang.String getPhone()
```

---


### setEmail

```
public void setEmail(java.lang.String string)
```

---


### setFirstName

```
public void setFirstName(java.lang.String string)
```

---


### setLastName

```
public void setLastName(java.lang.String string)
```

---


### setLoginName

```
public void setLoginName(java.lang.String string)
```

---


### getType

```
public java.lang.String getType()
```

---


### setType

```
public void setType(java.lang.String string)
```

---


### isComplete

```
public boolean isComplete()
```

---


### getLabGroup

```
public java.lang.String getLabGroup()
```

---


### getMessages

```
public java.lang.String getMessages()
```

---


### getPassword1

```
public java.lang.String getPassword1()
```

---


### getPassword2

```
public java.lang.String getPassword2()
```

---


### getSubmitterUserBean

```
public UserBean getSubmitterUserBean()
```

---


### setComplete

```
public void setComplete(boolean b)
```

---


### setLabGroup

```
public void setLabGroup(java.lang.String string)
```

---


### setMessages

```
public void setMessages(java.lang.String string)
```

---


### setPassword1

```
public void setPassword1(java.lang.String string)
```

---


### setPassword2

```
public void setPassword2(java.lang.String string)
```

---


### setPhone

```
public void setPhone(java.lang.String string)
```

---


### setSubmitterUserBean

```
public void setSubmitterUserBean(UserBean bean)
```

---


### getBioroot

```
public DBUtil getBioroot()
```

---


### setBioroot

```
public void setBioroot(DBUtil util)
```

---


### isExistingMember

```
public boolean isExistingMember()
```

---


### getDifferentUserConcat

```
public java.lang.String getDifferentUserConcat()
```

---


### setDifferentUserConcat

```
public void setDifferentUserConcat(java.lang.String differentUserConcat)
```

---


### getUserId

```
public int getUserId()
```

---


### setUserId

```
public void setUserId(int userId)
```

---


### setExistingMember

```
public void setExistingMember(boolean existingMember)
```

---


### isPasswordReset

```
public boolean isPasswordReset()
```

---


### setPasswordReset

```
public void setPasswordReset(boolean passwordReset)
```


---


|  |  |  |
| --- | --- | --- |
| **Overview** \| **Package** \| **Class** \| **Use** \| **Tree** \| **Deprecated** \| **Index** \| **Help** | |  |
| **PREV CLASS**   **NEXT CLASS** | **FRAMES**    **NO FRAMES**     **All Classes** |  |
| SUMMARY: NESTED \| FIELD \| CONSTR \| METHOD | DETAIL: FIELD \| CONSTR \| METHOD |  |


---
